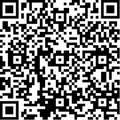# Implementation research and the HIV response: Taking stock and charting the way forward

**DOI:** 10.1002/jia2.26330

**Published:** 2024-07-05

**The articles in this supplement are designated by Washington University School of Medicine in St. Louis for AMA PRA Category 1 Credit™ for physicians. After reading the articles, access the accreditation information via the QR codes below**.

**Table:** 

Roy Paladhi U. et al. Effectiveness of HIV Self‐Testing when offered within Assisted Partner Services in Western Kenya (APS‐HIVST Study): A Cluster Randomized Controlled Trial	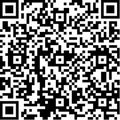
Sharma M. et al. Providing HIV assisted partner services to partners of partners in western Kenya: An implementation science study	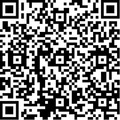
Nguyen VTT. et al. Investigating the effectiveness of web‐based HIV self‐test distribution and linkage to HIV treatment and PrEP among groups at elevated risk of HIV in Viet Nam provinces: a mixed methods analysis of implementation from pilot to scale‐up.	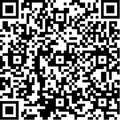
Vanhamel J. et al. Understanding adaptive responses in PrEP service delivery in Belgian HIV clinics: a multiple case study using an implementation science framework	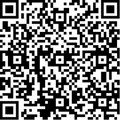
Nguyen N. et al. Long‐Acting Injectable ART to Advance Health Equity: A Descriptive Analysis of US Clinic Perspectives on Barriers, Needed Support, and Program Goals for Implementation from Applications to the ALAI UP Project	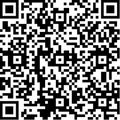
Chapuma C. et al. Examining barriers to antiretroviral therapy initiation in infants living with HIV in sub‐Saharan Africa despite the availability of point‐of‐care diagnostic testing: A narrative systematic review	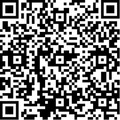
Mugambi LM. et al. Women's preferences for HIV prevention service delivery in pharmacies during pregnancy in Western Kenya: a discrete choice experiment	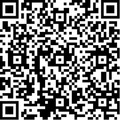
Velloza J. et al. Integrating a mental health intervention into PrEP services for South African young women: A human‐centered implementation research approach to intervention development	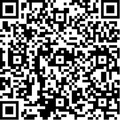
Tan BXH. et al. Fostering Citizen‐Engaged HIV Implementation Science	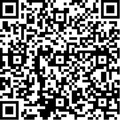
Chhun N. et al. Using FRAME to characterize provider‐identified adaptations to a stepped care intervention for adolescents and youth living with HIV in Kenya: A mixed methods approach	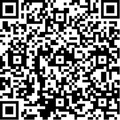
Enns B. et al. Estimating the potential value of MSM‐focused evidence‐based implementation interventions in three Ending the HIV Epidemic jurisdictions in the U.S.: a model‐based analysis	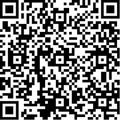
Lujintanon S. et al. Implementation strategies to improve HIV care cascade outcomes in low‐ and middle‐income countries: a systematic review from 2014 to 2021